# A277 A NOVEL PH-SENSITIVE Μ-OPIOID RECEPTOR AGONIST THAT DOES NOT INDUCE TOLERANCE IN A COLITIS MOUSE MODEL

**DOI:** 10.1093/jcag/gwac036.277

**Published:** 2023-03-07

**Authors:** C E Degro, N N Jiménez-Vargas, H M Schincariol, Q K Tsang, M Guzman-Rodriguez, A E Lomax, D E Reed, C Stein, N W Bunnett, S J Vanner

**Affiliations:** 1 Gastrointestinal Diseases Research Unit, Queen's University, Kingston, Canada; 2 Department of Experimental Anaesthesiology, Charité – Universitätsmedizin Berlin, Berlin, Germany; 3 Department of Molecular Pathobiology, New York University College of Dentistry, New York, United States

## Abstract

**Background:**

Adequate pain control in inflammatory bowel disease (IBD) can require opioids due to their high analgesic potency. The long-term use of opioids, however, is limited by the development of tolerance. This leads to reduced analgesic efficacy over time, resulting in escalating opioid dosing and thus increased risk of serious side effects. We previously demonstrated the safety and effectiveness of a novel pH-sensitive µ-opioid receptor (MOR) agonist, NFEPP, in a murine colitis pain model, but its tolerance potential with chronic administration is unknown.

**Purpose:**

To assess the tolerance potential of NFEPP compared to its parent compound fentanyl during acute colitis in a preclinical mouse model.

**Method:**

Acute colitis in C57BL/6 mice was induced using 2.5% dextran sulphate sodium for 5 days. NFEPP or fentanyl were then administered s.c. every 4 hours between 7am and 11pm over 5 days in daily increasing concentrations (0.4-1.5 mg/kg/d). Analgesic tolerance to opioids was assessed in conscious mice by measuring visceromotor responses (VMRs) to noxious colorectal distensions. Tolerance to the MOR agonist DAMGO in NFEPP or fentanyl treated mice was evaluated using patch-clamp recordings from dorsal root ganglion (DRG) neurons and extracellular recordings from lumbar splanchnic nerves that innervate the colon. Inflammation was assessed by macroscopic analyses, histological scoring and tissue-pH measurements of the inflamed colon. Group differences were analyzed using two-way ANOVA with Bonferroni′s or Tukey′s post-test (p-value <0.05).

**Result(s):**

NFEPP significantly reduced VMRs before and after chronic NFEPP treatment (39% reduction, p<0.05 vs. 41% reduction, p<0.05, compared to baseline at 80 µl). No differences of NFEPP induced antinociceptive actions were observed comparing VMR measurements before and after chronic administration (p=0.44). However, the analgesic activity of fentanyl decreased over time with less VMR inhibition observed after chronic treatment compared to fentanyl naïve mice (p<0.05). Cross tolerance testing between these two opioids revealed a loss of NFEPP- induced VMR inhibition in fentanyl treated mice, but fentanyl effects in the NFEPP treatment group remained unchanged. In patch-clamp recordings, DAMGO (100nM) evoked antinociceptive actions in DRG neurons from NFEPP treated mice (rheobase increase: 50%, p<0.05, compared to vehicle) whereas no change in rheobase with DAMGO was observed in DRG neurons from fentanyl treated mice. Afferent nerve activity from lumbar splanchnic nerves in response to von Frey filament probing decreased after DAMGO (100nM) application in NFEPP treated mice (p<0.05, compared to control probing), but showed no change in fentanyl treated mice. Colonic length, diameter, histological damage and tissue-pH did not differ between the two opioid treatment groups.

**Conclusion(s):**

Chronic administration of NFEPP during acute murine colitis does not lead to analgesic tolerance within the visceral nociceptive system, in contrast to its parent compound fentanyl.

**Please acknowledge all funding agencies by checking the applicable boxes below:**

CCC

**Disclosure of Interest:**

None Declared

